# Quantity implicature interpretations in bilingual population: the case of Imbabura Kichwa

**DOI:** 10.3389/fnhum.2024.1405373

**Published:** 2024-09-30

**Authors:** Santiago David Gualapuro Gualapuro

**Affiliations:** School of Languages and Linguistics, College of Liberal Arts, Southern Illinois University Carbondale, Carbondale, IL, United States

**Keywords:** Kichwa, *wakin*, some, pragmatic interpretation, *algunos*, indigenous language, pragmatic interpretation bilingual

## Abstract

Most studies on the pragmatic interpretation of existential quantifiers have been conducted in major Indo-European languages like English, Spanish, French, and Greek, focusing mainly on monolingual participants. However, in indigenous linguistic research, especially experimental research, it is crucial to consider several linguistic and extra-linguistic factors for successful implementation. Our research centered on the experimental investigation of the pragmatic interpretation of the quantifier *wakin*, meaning *some* in Kichwa, with Kichwa-Spanish bilingual adults from the province of Imbabura Ecuador. We employed the Truth Value Judgement Task (TVJT) for our experiments and incorporated the explicit Question Under Discussion (QUD) paradigm to facilitate pragmatic interpretations among our participants. Our initial experiment revealed a 78% acceptance level for the pragmatic interpretation of “some, but not all” in Kichwa, significantly lower than the 95% acceptance range observed in other languages. We hypothesized that access to technology and formal education might influence these results, leading us to simplify our experiment by eliminating the technological components of the research. In our subsequent experiment, adult speakers of Imbabura Kichwa achieved a 97% accuracy level, comparable to speakers of other languages. To benchmark our results against speakers of other languages under similar conditions, we evaluated whether Spanish speakers from two varieties in Ecuador (Quito and Guayaquil) could generate the “some, but not all” scalar implicature with the Spanish quantifier *algunos*. Our findings indicated that speakers achieved 95.5 and 97.4% accuracy for both varieties, respectively. Therefore, this study infers that under optimal conditions, speakers of indigenous languages in rural communities demonstrate commendable performance in experimental linguistic studies. Nonetheless, it underscores the necessity for meticulous planning and distinct handling in experimental studies involving speakers of these languages residing in rural areas without access to technological elements. We propose that such research broadens our comprehension of language utilization in minority communities and positively influences language restoration efforts by expanding experimental linguistics studies to indigenous languages.

## 1 Introduction

Few pragmatic interpretation of scalar implicature studies have been done with settled adult bilingual populations (Dupuy et al., 2018; Antoniou and Katsos, 2017; Slabakova, 2010). Most of these studies have argued, though not conclusively, that bilingual speakers possess advantages in pragmatic interpretation over monolingual speakers. These studies of pragmatic interpretation of existential quantifiers thus far, have been done in major Indo-European child and adult languages, including English *some* (Contemori et al., 2018; Miller et al., 2005; Smith, 1980); Spanish *algunos* (Pratt et al., 2018; Grinstead et al., 2010; Vargas-Tokuda et al., 2009, 2008; Lopez-Palma, 2007); French *certain* (Noveck, 2001); Greek *meriki* (Papafragou and Musolino, 2003); and Dutch *somige* (de Hoop, 1995), focusing on pragmatic production by monolingual participants. Furthermore, none of these studies has been carried out in an indigenous language of the Americas. Imbabura Kichwa is an indigenous language spoken in the Northern Andean province of Imbabura in Ecuador. Kichwa, or Imbabura Kichwa, is a Quechuan language variety spoken in the northern province of Imbabura in Ecuador (Gualapuro, 2017; Adelaar, 2004; Muysken, 1992; Cole, 1982).

The structure of this article offers a concise analysis of the pragmatic interpretation of the existential quantifier *some* and its equivalent existential quantifiers in other languages, such as *algunos* in Spanish. This study also provides supporting literature regarding studies on the pragmatic interpretation of *some* in bilingual contexts and the cognitive factors driving it. It introduces the Kichwa existential quantifier *wakin* and outlines a detailed research methodology, reporting findings from four unique experiments. The first experiment explores the use of *wakin* among Kichwa-Spanish bilingual speakers in rural areas near Cotacachi and Otavalo in Imbabura. The subsequent two experiments scrutinize the Spanish quantifier *algunos* among two demographics: Kichwa-Spanish bilinguals and Spanish monolinguals from Cotacachi and Otavalo, and Spanish monolinguals from Quito and Guayaquil, respectively. The final experiment focuses on *wakin*, with highly educated Kichwa-Spanish bilinguals with substantial technological skills and urban lifestyles. The study culminates in an exhaustive discussion and conclusion of the findings.

### 1.1 Pragmatic implicature

Grice (1975, p. 43) states that quantity implicatures are generated due to a quantity scale existing in the lexicon. This scale means that the utterance of a given value on a scale will implicate that “as far as the speaker knows, no higher value applies. This scale includes the quantifiers *all, many, some, few*, and *none*. In conversational settings, reading between the lines, the quantifier *some* gets the ‘some, but not all' meaning by being less informative than *all* (Horn, 1972, p. 70–154).” In other words, if someone says a phrase as in (1), the message implies s/he did not eat all of the cookies.

(1) I ate *some* cokies.

This study focuses on the “some, but not all” reading; however, *some* is a pluri-functional quantifier or can be associated with meanings other than the focus reading of this study. Alternative interpretations include using some to refer to someone or something unknown, unspecified, or pragmatically non-identified.

(2) I talked to some doctor the other day, but I do not
remember who.

Sentence (2) is unspecified, unknown, and also pragmatically non-identified. These properties of *some* provide a door to study them further; however, it is not the topic of interest of this study.

This “some, but not all” interpretation of the noun phrase (NP) is taken to be the result of higher-order reasoning, making an inference in a particular syntactic and pragmatic context, producing an interpretation that Grice (1975, p. 45) referred to as a “scalar, generalized conversational implicature.” The example in (3) below illustrates this interpretation.

(3) *Some* students passed the exam.

In other syntactic and/or pragmatic contexts, the same noun phrase (*some* students) can be understood to mean “some, and possibly all”, as in this example 4.

(4) If *some* students come to my office hours, I'll buy you
lunch.

In sentence (4) the “*some, but not all”* pragmatic meaning has disappeared and been replaced with the “some, and possibly all” logical meaning, which occurs in syntactic contexts (known as Downward Entailing contexts) such as the antecedent clause of a conditional sentence (Ladusaw, 1979).

### 1.2 Pragmatic interpretation of quantifiers in English

Since Smith (1980) first investigated the quantification of *some* with English monolingual adults and children, results have told us that adults and children can (on various degrees of acceptance) read the “some, but not all” quantification with the English quantifier *some*. Though Smith (1980) did not set out to study pragmatic implicature generation or cancellation, it became the first experimental study in quantifiers in English. Her experimental context constituted a downward-entailing context (yes-no questions) in the sense of Ladusaw (1979), which resulted in the logical “some, and possibly all” interpretation of the existential quantifier *some* being prominent.

In English, it seems to be the case that the phonetic properties of some words play an essential role in their interpretation. Miller et al. (2005) found that the phonetic properties of some could play an important role in shedding the pragmatic interpretation of *some*. They found that the contrast between (5a) and (5b) below allows a construction of sentences that both contain a weak quantifier but differ in the presence or absence of a quantity implicature. In sentences like (5b), if *some* is the stressed form *SOME*, the scalar implicature effect caused by the pitch-accented version seems to be implemented more strongly. Miller et al. (2005) state that “the focus (by the stress) on the quantifier induces the scale as the alternative set.”

(5) a. Make *some* happy faces
b. Make *SOME* faces happy

Grinstead et al. (2010) and Thorward (2009) found that *some* have three phonetically different forms, including the syllabic nasal *sm*, the full vowel no pitch-accented *some*, and the full vowel, pitch-accented *SOME* (see Table 1). Thorward (2009) demonstrated that the three forms of the quantifier some has the following interpretations: the weak form *sm* with a purely existential interpretation similar to *unos* in Spanish; the pitch accented *SOME* that generates the implicature of “some, but not all” and “some, and possibly all”; and the bare form *some* similar to *algunos* in Spanish but functioning strictly as a pure quantifier (Grinstead et al., 2010; Thorward, 2009). These variants of *some* have specific properties concerning word duration, vowel duration, and pitch. Thorward (2009) gave the following means of these variables for each type of *some* tested.

**Table 1:** Properties of *some* (Grinstead et al., 2010; Thorward, 2009).

**Type of *some***	**Word duration**	**Vowel duration**	**Maximum Pitch (Hz)**
*sm*	0.301	–	297.7
*some*	0.350	0.139	273.2
*SOME*	0.398	0.154	471.2

Experimentally, for implicature generating contexts, Grinstead et al. (2010) showed that English-speaking adults were able to distinguish differences between the *SOME* and *some* conditions (χ^2^ = 5.4, *p* = 0.02) as well as between the *SOME* and *sm* conditions (χ^2^= 15.8, *p* < 0.01). Their results also showed significant difference between *sm* and *some* (χ^2^ = 10.5, *p* = 0.001) and between *sm* and *SOME* (χ^2^ = 19.5, *p* < 0.001) but not between *SOME* and *some*. These results showed that adults “rely heavily on the presence or absence of contrastive pitch accents” to calculate a “some but not all” implicature meaning in English. This study also included children. Grinstead and colleagues found that children can deliver the “some but not all” pragmatic interpretation. However, children rely heavily on cues from vowel realization and do not rely significantly on the placement of contrastive pitch accents. The current work (built on Grinstead and colleagues's findings) explores the possibility of a pitch-accented effect in Kichwa's quantifier *wakin*. It is expected that the final syllable stressed form [wa.ˈkiŋ] (instead of the canonical penultimate syllable stressed form [ˈwa.kiŋ]) of *wakin* is the form to generate the implicature interpretation.

### 1.3 Pragmatic interpretation of quantifiers in Spanish

In Spanish there are two *some-like* equivalent existential quantifiers; *algunos* and *unos* (Gutiérrez-Rexach, 2004, 2001). Gutiérrez-Rexach (2001) notes that “there are significant differences between *unos* and *algunos*. The crucial distinction relevant to this study is that *unos ‘a-pl'* cannot occur as the subject of individual level predicates, whereas *algunos ‘some-pl'* can Gutiérrez-Rexach (2001, p. 118).” In this line, Vargas-Tokuda et al. (2008) considers that *algunos* allows a “*some, but not all”* pragmatic implicature, which “can be canceled in downward entailing environments,” created by the antecedent of a conditional.

The existential quantifier *unos* appears to “permit truth-conditionally similar *some, but not other*” interpretations according to Pratt et al. (2018). However, she also states that “through its *unos…, otros no* articulation,” a collective reading. She argues that *unos* cannot generate the “some, but not all” interpretation, which aligns with the *unos* properties as described in Gutiérrez-Rexach (2001)'s studies.

Using the Truth Value Judgement Task method, Vargas-Tokuda et al. (2009) tested 27 monolingual, Spanish-speaking children (Age Range = 4.9–6.7, *M* = 5.9) from a daycare center in Mexico City, and 10 monolingual, Spanish-speaking adults from the same city. These children and adults could generate the quantity implicature with *algunos* sentences as in (6) where X are animals.

(6) *Algunos* X saltaron sobre A
*Some*-A Xs jumped over A

Her experiment showed that Spanish-speaking adults (80%) could distinguish the differences between *unos* and *algunos*. She also found that children “can distinguish *unos* and *algunos* at a young age” at the 70% rate (Vargas-Tokuda et al., 2009). These results show that monolingual Spanish-speaking adults could deliver the “some, but not all” pragmatic interpretation with *algunos*. In contrast to Noveck (2001), they also argue that even 5-year-old children in this study could generate a “*some, but not all*” pragmatic implicature with *algunos*.

Pratt et al. (2018) also conducted a more extensive (than Vargas-Tokuda's) experimental design for *algunos* and *unos* in Mexico City with 60 college students who were monolingual Spanish-speaking adults (Range: 220–445 months, *M* = 305.5 months, *SD* = 63 months) and 42 typical developing children (Range: 61–84 months, *M* = 69.74 months, *SD* = 5.42). Their experiments used two Question Under Discussion (QUD) conditions, *quiénes* (who) and *cuántos* (how many), under the Truth Value Judgment Task (TVJT) approach (Crain and Thornton, 1998). Results for both QUD forms showed that for *quiénes*, adult college students generated implicature readings 100% of the time. For *cuántos*, college student adults generated the implicature reading 94% of the time. Pratt et al. (2018) and Vargas-Tokuda et al. (2009) experiments showed that monolingual Spanish-speaking adults in Mexico City could generate the “some, but not all” reading with the Spanish existential quantifier *algunos*. The stimuli applied in Pratt et al. (2018)'s research were adapted to the Ecuadorean Spanish context in this project for experiments 2 and 4. This adaptation is a crucial step in ensuring the relevance and applicability of the experiment to the specific linguistic context of Ecuador.

### 1.4 Pragmatic interpretation of quantifiers in other languages

Research initiatives focusing on the pragmatic interpretation of quantifiers in languages other than English or Spanish are relatively scarce. One such study was conducted by Noveck (2001), who examined the production of scalar implicatures of the existential quantifier *certain* (*some*) among French-speaking children and adults. The results of Noveck's experiment indicated that adults delivered the “some, but not all” interpretation 87% of the time, while children achieved this interpretation 41% of the time. This led Noveck to conclude that children's ability to generate implicature interpretations in French was not yet fully developed in an adult-like way in French.

In a separate study, Papafragou and Musolino (2003) tested Greek children and adults on the interpretation of the Greek quantifier *meriki* meaning *(some)* in English. Ten Greek children, five years old (*M*: 5.3 years old) and 10 adults participated in this experiment for *meriki*. They used the following type of sentences in (7) as stimuli.

(7) *Merika* apo ta aloga pidiksan pano apo to fraxti
“ *Some* horses jumped over of the fence”

Papafragou and Musolino (2003) found that adults generated implicature 92.5% of the time, while children generated the implicature only 12.5% of the time with the Greek quantifier *meriki*. Based on these findings, they proposed that preschoolers, and children in general, do not appear to be as sensitive to the pragmatic interpretation of scalar terms as adults.

Both studies (Papafragou and Musolino, 2003; Noveck, 2001), demonstrated that adult speakers of other languages (French and Greek, in addition to English and Spanish) were capable of generating the “some, but not all” scalar implicature with their language's equivalent of *some*. It is anticipated that this experimental study involving Kichwa adults will also yield the “some, but not all” implicature interpretation with the Kichwa quantifier *wakin*. This forthcoming experiment has the potential to enrich the existing body of research on the pragmatics of quantifiers in languages that are not traditionally studied.

### 1.5 Bilingualism and cognition in scalar implicature reading

Sociolinguistic variables can become predictors of linguistic knowledge, interpretation and social distinction, (Eckert, 2012; Labov, 1972, 1966). Education level, linguistic background, and access to technology are some of the critical variables studied in language production (Dupuy et al., 2018; Syrett et al., 2016, 2017; Slabakova, 2010).

The relevance theory postulated by Sperber and Wilson (1995) states that interpreting implicatures involves extra cognitive effort, in opposition to the literal meaning mentioned by Antoniou and Katsos (2017); Prior (2012) and Pouscoulous et al. (2007). Studies have suggested opposing results (positive and negative) for the effects of bilingualism on cognitive functioning (Cockcroft et al., 2019; Contemori et al., 2018; Antoniou and Katsos, 2017; Marton et al., 2017; Bialystok, 2015, 2001; Barac and Bialystok, 2012). Language production and cognitive abilities in monolinguals and bilinguals have been studied thoroughly; however, Antoniou and Katsos (2017, p. 2) mention that “pragmatic-communicative skills have so far received little attention”.

Siegal et al. (2007) tested Italian-Slovenian bilingual children's interpretation of scalar implicature in Italian. In this test, bilingual children also performed better than monolingual children. They also repeated the test with Italian-German bilinguals in Italian in 2010 and obtained similar results; bilingual children performed better than monolinguals. Further, Siegal et al. (2007) explored whether bilingualism confers an advantage to pragmatic competence in bilingual Japanese-English children. Their experiments showed that 6-year-old Japanese-English bilingual children were more advanced in their scalar implicature interpretation in Japanese than their Japanese monolingual peers. All their experiments showed that bilingual children in different languages better understood pragmatic implicature interpretations than their monolingual peers.

On the other hand, Dupuy et al. (2018) showed mixed results in studying adult native speakers of French and L2 learners of either Spanish or English in scalar implicature reading. French speakers who were Spanish and English L2 learners showed better performance in pragmatic interpretation than French monolingual peers, *p* < 0.05, but no differences between the L2 languages, *p* > 0.05. Comparing between English and Spanish L2 learners, they showed no differences in their pragmatic interpretations, *p* > 0.05. Furthermore, in their experiment, studying whether there is a genuine increase of pragmatic abilities when learning an L2 language, under a between-subjects design, L2 learner did not show better performance than the French monolingual peers, *p* > 0.05.

Syrett et al. (2017) also found that children and young adult Spanish-English bilinguals in NJ, USA, performed well in their pragmatic interpretation tasks in Spanish. However, they did not find a bilingual advantage in scalar implicature reading in contrast to other experimental findings (Siegal et al., 2010, 2009, 2007). Even more Syrett et al. (2017, p. 16) argue that bilingual children may face even more significant difficulties in “successfully distinguish specific lexical entries from each other (language)”. According to Syrett et al. (2017) these difficulties are compensated as children grow and proficiency in their language(s) increases.

The experimental results analyzed here show us that there is no clear consensus of whether or not bilingual speakers have cognitive advantages in pragmatic reading. In order to grasp linguistic and extra-linguistic factors that can influence pragmatic reading, Antoniou and Katsos (2017) used vocabulary tests such as the Word Finding Expressive Vocabulary Test and the Wechsler Abbreviated Scale of Intelligence (WASI) test. They also performed the Alberta Language Environment Question (ALEQ), (Paradis, 2011), the Alberta Language Development Questionnaire (ALDeQ) (Paradis et al., 2015). The Socio-Economic-Status (SES) variables measured for their experiment showed no correlation in pragmatic interpretation. Antoniou and Katsos (2017) found that age was a significant predictor of pragmatic interpretation in multilingual groups but not for monolinguals. Their findings suggest that other “non-linguistic factors, besides Executive Components (EC), that develop with age are possibly involved in and sustain the process of computing implicatures in multilinguals,” but failed to mention what they are.

The current research endeavor investigates the potential influence of sociolinguistic and extra-linguistic factors, including education level, Language Use, L1 proficiency, computer access, and internet access can have an impact on the pragmatic interpretation of the Kichwa quantifier *wakin* in Imbabura Kichwa. All these variables were captured using the ALEQ-3 questionnaire Soto-Corominas et al. (2020) adapted to the Imbabura Kichwa context. Drawing upon prior studies conducted in bilingual populations, there is no conclusive evidence to suggest that bilingualism confers any benefits or advantages in tasks involving pragmatic interpretation. The current research project was conducted within a Kichwa-Spanish bilingual context. Consequently, it is anticipated that bilingualism may exert some influence on our findings. Our results could shed further light on whether bilingualism confers an advantage in pragmatic interpretation within the Kichwa-Spanish bilingual context.

### 1.6 Kichwa quantifier *wakin*

Imbabura Kichwa quantifiers are *tukuy* = *all, wakin* =*some* and *tawka* = *many*. Negative quantifiers are *mana ima* shortened to *nima* = *none* or *nothing* as influenced by Spanish contact with an insertion of the Spanish negative conjuction *ni* meaning “*not even*”. For Southern Quechua, Muysken (1992) and Faller and Hastings (2008) give some details of the properties of the Quechua quantifiers:

(a) Quantifiers, morphologically nouns, can be inflected for person and number.
(b) Quantifiers may be “floated away” from the element they modify.
(c) Quantifiers differ in the extent to which they trigger subject or object agreement on the verb.

Muysken (1992) also noted that Southern Quechua quantifiers carry inflectional markers. He states that there are three different forms to be distinguished: (a) obligatory inflection, (b) optional inflection, and (c) no inflection. He argues that the indefinite quantifier *wakin* “can, but need not carry person marking.” In many dialects of Quechua, *wakin* is an obligatorily inflected quantifier. Faller and Hastings (2008) argue that while “quantifiers can occur prenominally, they often also occur without a head noun” in Quechua. They also classiffy *wakin* as a strong quantifier among the other Quechua quantifiers. Faller and Hastings (2008) use these terms in a purely descriptive manner, arguing that weak quantifiers can occur in existential sentences, and strong ones are those excluded from this environment. Sentences (8) to (11) show the possible scenarios where *wakin* may occur in Imbabura Kichwa.

(8) *Wakin* runa-kuna ri-naku-n
*some* people-PL go-PL.PROG-3
*Some* people are going xlist
(9) *Wakin-kuna runa-kuna ri-naku-n
*some-PL people-PL go-PL.PROG-3
*Some people are going xlist
(10) *Wakin-kuna* ∅ ri-naku-n
*some-PL* ∅ go-PL.PROG-3
*Some* (people) are going xlist
(11) *Wakin* ∅ ri-naku-n
*some* ∅ go-PL.PROG-3
*Some* (people) are going

In example (8), *wakin* stands alone with no inflectional morphemes attached. In the case of Imbabura Kichwa, this is the canonical form where *wakin* occurs, and this is the form used for the experimental process in this study. Moving forward, example (9) does not allow double inflection, turning the example ungrammatical. Imbabura Kichwa does not allow double plural marking at the NP node.

Example (10) is grammatical even though it does not have the head noun. In this example, we presuppose that the object we refer to (people) exists in the discourse and was previously introduced to the audience. Incorporating a noun into sentence (10) renders the sentence ungrammatical.

In (11), the plural marker *-kuna*, is not required as in *wakin rinakun*, meaning (some (people) are going); *wakin* already gives the reading of more than one interpretation. According to Faller and Hastings (2008) and Muysken (1992), *wakin* is presupositional, already discussed in sentences (10) and (11). Faller and Hastings (2008) argue that “*wakin*-quantified noun phrases are felicitous only in contexts where the non-emptiness of the restriction is presupposed.” The previous statement assumes that the elements (plural) are known to the speakers, and they are aware of it, as we explained in sentences (10) and (11). To support this claim, Faller and Hastings give an example for Cuzco Quechua in (12) and (13) below, where *wakin* can be optional, and is simply understood conversationally as *some*:

(12) Tari-sqa-ku-raq (*wakin*) dodo-kuna-ta
find-NX.PST-PL-CONT some dodo-PL-ACC
“ They found (*some*) dodos.”
(13) *Wakin* loro-kuna rima-nku
some parrot-PL talk-3PL
“*Some* parrots talk” (and others are presumed not to talk)

The provided examples, particularly example (12) offer compelling evidence that the inflection of the indefinite quantifier *wakin* in Cuzco Quechua is optional and context-dependent. This is consistent with *wakin*'s property as an indefinite yet proportional quantifier. Faller and Hastings (2008) state that *wakin* “is therefore more similar to the English stressed *SOME* than the partitive some of (the).”

To this point, no experimental studies have been conducted on pragmatic interpretation in Indigenous languages, as previously discussed. Consequently, this paper aims to experimentally ascertain whether the Imbabura Kichwa quantifier *wakin* permits the pragmatic scalar interpretation of “wakin, mana tukuy = some, but not all,” as observed in other languages. To address this query, this paper presents a discussion of the four experiments executed for this purpose. Each experimental unit is unique, and as a result, the research questions are provided prior to each experimental procedure, and the analysis of the results is given after each experiment. This approach guarantees a comprehensive understanding and seamless flow between each experiment and permits a methodical evaluation of each one. This strategy reinforces the consistency of the paper and promotes a structured examination of each experiment.

## 2 Experiment 1

Imbabura Kichwa (as well as the Ecuadorean Kichwa language family) does not strongly prefer stress-accented solid forms in the language (Lombeida-Naranjo, 1976; Cole, 1982). However, as a native speaker of Imbabura Kichwa, I have experienced the use of stress to emphasize the topic being discussed in a casual conversation. As in English, I consider that there is an accented form of the Kichwa quantifier *wakin* that delivers the “some, but not all” pragmatic interpretation. Following Faller and Hastings (2008)' statement that Kichwa's *wakin* is similar to the pitch-accented *SOME* in English, I aim to investigate the following research questions.

1. Do Kichwa speakers recognize two variants of the quantifier *wakin*: the canonically accented [ˈwa.kiŋ] and the final syllable accented form [wa.ˈkiŋ]?
2. Do Imbabura Kichwa-Spanish bilingual adult speakers generate the “wakin, mana tukuy = some, but not all” pragmatic interpretation with the Kichwa existential quantifier *wakin* under the Truth Value Judgement Task using the explicit Question Under Discussion paradigms with either variant of *wakin*?

### 2.1 Participants

For this first experiment, a total *N* = 18 adult Kichwa-Spanish bilingual speakers from the indigenous communities of Gualapuro in Otavalo and El Cercado in Cotacachi were selected—participants who did not pass the fillers (*N* = 6) were not included for statistical procedures. A total of 12 participants (*M* = 35.5 years, SDev = 13.7 years, Range: 19.4–58.4 years) who passed the fillers stimuli were selected for statistic measures. All our participants in this experiment were early sequential bilinguals (Amengual, 2019), they were exposed to Kichwa at birth and learned Spanish at school or later in life.

Table 2 shows the descriptive statistics for sociolinguistic variables from the ALEQ questionnaire.

**Table 2:** ALEQ results.

**Category**	**Mean**	**Standard dev**.	**Range**
Age (in years)	35.53	13.71	18–58.4
Education years	6	3.84	0–12
Computer use	1.33	1.46	1–3
Internet access	1.12	1.36	1–3
Kichwa fluency	4.83	–	1–5
Language use	1.4	0.98	1–5
Language ability	2.43	0.91	1–5

All ALEQ-3 (Soto-Corominas et al., 2020) variable averages come from participants' self-reported data. The results from the ALEQ-3 questionnaire show us that participants in this first experiment were older than any of the participants in previous pragmatic experiments regarding quantifiers (Dupuy et al., 2018; Siegal et al., 2010, 2009; Syrett et al., 2017; Pratt et al., 2018; Janssens et al., 2014). We also observe that the Education Years (*M* = 6 years) are very low compared to adult participants in other language experiments. We measured other sociolinguistic variables like Computer Use. The scores were computed as follows: 1 = no Computer Use; 2 = sometimes; 3 = always, averaging 1.3 (see Table 2). This means that the average participants in this experiment do not know how to use a computer. Also, using the same scale (1–3), results showed that most of our participants did not have Internet access in their homes.

When talking about Fluency in Kichwa, the results (*M* = 4.83, Range 1–5) show us that they are completely fluent in the language. They use the language in most of their daily communication with others in rural areas.

Language Use was measured by asking which language participants used the most with their close and extended family members. Scores were given according to this criterion: Only Kichwa = 1; More Kichwa Less Spanish = 2; Equal Kichwa and Spanish = 3, More Spanish Less Kichwa = 4, Only Spanish = 5. Results for Language Use show us that our participants were communicating almost all the time in Kichwa (*M* = 1.4).

We also measured the Language Ability of the participants. Participants self-reported their ability to read, write, listen, and understand both languages, Kichwa and Spanish, using the following scale: Only Kichwa = 1; More Kichwa Less Spanish = 2; Equal Kichwa and Spanish = 3; More Spanish Less Kichwa = 4; Only Spanish = 5. Overall, the results from Table 2 show us that participants are more or less balanced bilinguals leaning toward Kichwa dominance (*M* = 2.43, Range = 1–5). However, Kichwa is not taught in schools (Conejo, 2008; Haboud, 1998) but rather is orally transmitted. Consequently, we needed to analyze this data more closely (see Table 3).

**Table 3:** Language ability.

**Category**	***N***	***Mean***	***Standard dev*.**	***Range***
Speaking	12	2.50	.905	1–5
Listening	12	2.833	1.15	1–5
Reading	12	3.42	1.31	1–5
Writing	12	3.50	1.679	1–5

We performed a paired *t*-test comparing the means for these variables. The Speaking-Listening paired *t*-test showed no difference in scores among them, [*t*(11) = −1.483, *p* = 0.166]. Similarly, there was no difference in scores among the Reading and Writing variables, paired *t*-test, [*t*_(11)_ = −0.290, *p* = 0.777]. There are significant differences in scores between Speaking and Reading, [*t*_(11)_ = −4.750, *p* = 0.001]; Speaking and Writing, [*t*_(11)_ = −25.71, *p* = 0.02]; Listening and Reading, [*t*_(11) = −3.02, *p* = 0.12], and no significant differences in Listening and Writing, [*t*_(11)_ = −2.00, *p* = 0.07]. We think these results are valuable for understanding Kichwa's situation as a traditionally oral language.

Performing a co-linearity test, the variables Access to the Internet (VIF = 5.33) and Computer Use (VIF = 7.37) showed high co-linearity effects for the Education Years variable. Therefore, the Computer Use variable was chosen for the statistical calculations.

### 2.2 Procedures

The stimuli discourse units used for this experiment were translations of the Spanish version used in Mexico City by Grinstead et al. (2010) to the Kichwa language. Four warm-ups, four fillers, and ten experimental sentences were developed under the criteria of Question Under Discussion (QUD) conversational discourse (Roberts, 2003). For the experimental stimuli, ten discourse sentences were created for each phonetic variant, [ˈwa.kiŋ] and [wa.ˈkiŋ]. They were created and assembled in “3 out of 4” and “4 out of 4” contexts of the stimuli. These contexts were set in the idea that “3 out of 4” and “4 out of 4” cartoon characters are performing an action in a motion-animated audio and video scenario.

### 2.3 Stimuli

For this experiment, we used a variation of the traditional TVJT in Mexican Spanish, as first implemented by Grinstead et al. (2019). The audio content was later translated into Kichwa and recorded by a Kichwa-Spanish-English tri-lingual female speaker using Audacity v.2.4.1 directly into a personal computer, which allowed experimenters to control some aspects of the context, including Prosody, Conversational Common Ground (CCG), and Question Under Discussion (QUD). The reader was a native speaker of Imbabura Kichwa and a native speaker of Highland Ecuadorean Spanish. Here is one of the experimental stimuli examples.

(14) Wawa-kuna-ka wasi-pi-mi television-ta riku-nkapak
chakana-ta witsiya-nkapak muna-n.
child-PL-TOP house-LOC-AFF tv-ACC watch-COREF ladder-ACC climb- COREF want-INF-PROG.
“The children want to climb the ladder to go watch TV.”

Sentence (14) creates a background information that sets the stage for the upcoming QUD scenario. The following sentence (15) gives more detailed information about the activity these cartoon characters are about to perform.

(15) Uh chakana-ka yapa hatun-mari mana tukuy-lla
witsiya-y ushan-ka-chu yani-mi.
Uh-Oh ladder-TOP much high-EMPH-witness NEG all-LIM climb-IMP can-3FUT-DUB think-VAL
“Uh-Oh! The ladder is too high, I don't think all the children will be able to climb it.”

Here in (16), we have the explicit Question Under Discussion scenario which prepares participants for the YES/NO answer to the upcoming question.

(16) Pikuna-shi witsiya-nka chakana-ta?
who-PL-REP  climb-3FUT  ladder-ACC-TOP.QUESTION
“Who is going to climb the ladder?”

This sentence (17) contains our target quantifier, in which participants are expected to answer YES to the “3 out of 4” context and reject the “4 out of 4 context” for all the experimental stimuli for both of the forms of the quantifier *wakin*.

(17) Ahh ña yacha-ni [ˈwa.kiŋ/wa.ˈkiŋ] wawa-kuna
witsiya-rka chakana-ta
well now know-1 some child-PL climb-PAST ladder-ACC
“Well, I know. *Some* children were able to climb the ladder”

The speaker was instructed to pronounce *wakin* as [ˈwa.kiŋ] and [wa.ˈkiŋ] for each stimuli. These audio recordings were then analyzed for pitch, duration, and intensity for both [ˈwa.kiŋ] and [wa.ˈkiŋ] at the last syllable using the Praat v.6.0.4 software. Table 4 shows the results of these measurements.

**Table 4:** Phonetic variants of *wakin*.

	ˈwa.kiŋ			wa.ˈkiŋ		
	**Pitch**	**Intensity**	**Duration**	**Pitch**	**Intensity**	**Duration**
Last-syllable (mean)	300.9	78.9	282.7	315.25	82.5	472.17
Standard dev.	67.3	1.86	82.3	76.64	2.45	32.69

Pitch in (Hz), intensity in (dB), duration in (ms).

Running the R software, the One-way ANOVA test found that pitch on the final syllable was not significantly different for the two forms of *wakin* [*F*_(1, 18)_ = 0.60, *p* = 0.66]. For intensity, the One-Way ANOVA test showed an effect on the final syllable intensity on quantifiers [*F*_(1, 18)_ = 13.4, *p* = 0.002]. The same One-Way ANOVA test showed an effect of final syllable duration on the quantifiers [*F*_(1, 18)_ = 45.69, *p* < 0.001]. For this experiment, we created two forms of the quantifier, [ˈwa.kiŋ] and [wa.ˈkiŋ] which differed in Duration and Intensity but not pitch. These stimuli and the warm-ups and fillers were encoded in the SuperLab v5 software. The four warm-up stimuli were placed at the beginning of the trials to familiarize participants with the upcoming experimental work. The experimental and filler stimuli were placed in a random order. Participants were distributed evenly under the between-subjects experimental design in which the subjects are assigned to different conditions.

Before starting the experiments, all participants were asked to complete the adolescents (adult) version of the Alberta Language Evaluation Questionnaire (ALEQ) by Soto-Corominas et al. (2020). Only filler passer's ALEQ results are included for statistical measures.

For the experimental part, participants were asked to press the C key in the keyboard which was labeled with a happy face 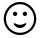 if they agree with the statement or press the M key labeled with a sad face 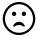 if they disagree with the statement.

Reaction Time (RT) (in milliseconds = ms) was also calculated following this criterion. The duration (in ms) from the beginning of the stimuli up to the beginning of the quantifier pronunciation was calculated. This time was called Time Before Quantifier (TBQ). A second measurement was also calculated from the beginning of the audio stimuli until the participants pushed the key, called End Point Time EPT. Then, using simple mathematical operations, the RT was calculated as the difference of EPT−TBQ.


RT=EPT−TBQ

### 2.4 Results

#### 2.4.1 Descriptive statistics

In the following table, we have the means for acceptance for the 3 out of 4 and 4 out of 4 contexts for both forms [ˈwa.kiŋ] and [wa.ˈkiŋ] of the Kichwa quantifier *wakin*. A total of *N* = 6 participants, three for each phonetic variant of *wakin*, were discarded from the sample. These participants failed to answer correctly the filler stimuli (see Table 5).

**Table 5:** Acceptance rate by quantifier form.

**Context**	**Quantifier forms**				**Total**	
	**WAkin**	**%**	**waKIN**	**%**	**Acceptance**	**%**
3 out of 4	26/30	86.67%	25/30	83.33%	51/60	85%
4 out of 4	9/30	30%	4/30	13.33%	13/60	21.67%

#### 2.4.2 Inferential statistics

**Figure 1:**
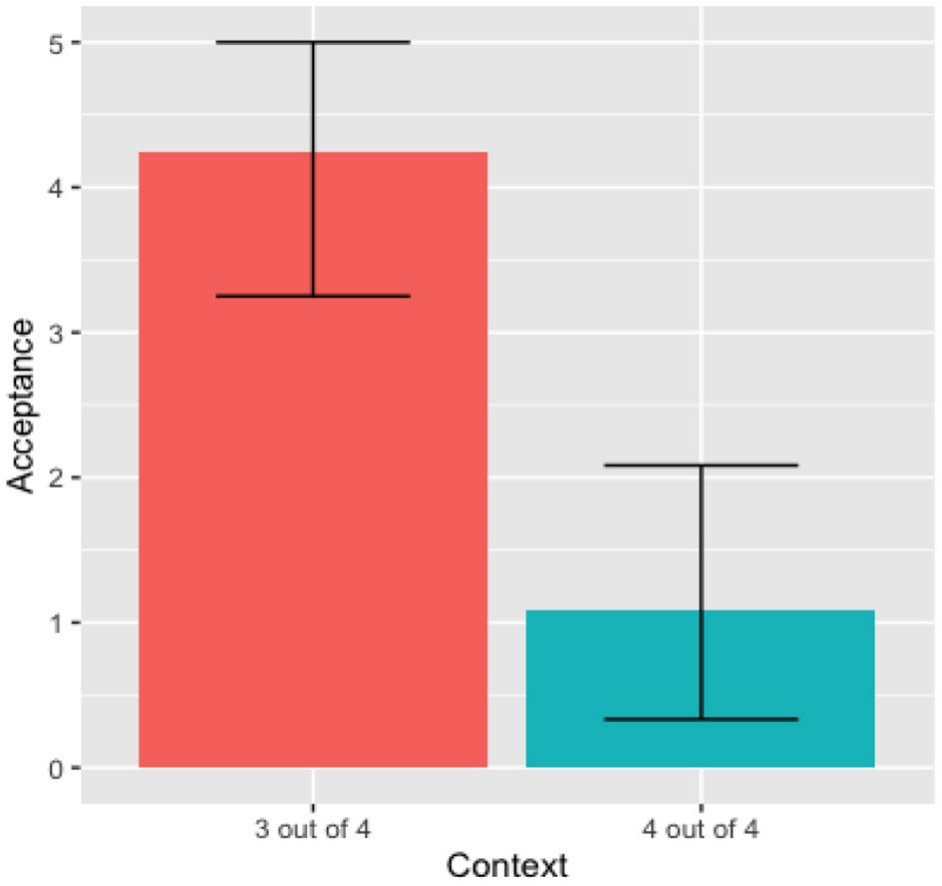
Adults' mean acceptance *wakin* (condensed).

In calculating the inferential statistics, we use the R version 1.3.959 software. The two-way ANOVA test, considering Context as a function of Acceptance and the Phonetic Forms of the quantifier, showed no significant effect of the phonetic forms in the Acceptance rate [*F*_(1, 21)_ = 0.29, *p* = 0.60]. Looking into each context, the One-way ANOVA test for the “4 out of 4” context, show no significant effects of the two phonetic forms in the Acceptance rate, [*F*_(1, 10)_ = 0.776, *p* = 0.399]. Similarly, the One-way ANOVA test for the “3 out of 4” context shows no significant effects of the phonetic forms in the Acceptance rate [*F*_(1, 10)_ = 0.03, *p* = 0.867]. These results show that Kichwa speakers do not recognize different phonetic variants for the quantifier *wakin* for the tested phonetic variables (intensity, pitch, and duration). Thus, there was no difference between quantifier variants nor between contexts. Consequently, this data was condensed into a single distribution for the quantifier *wakin*, as observed in Figure 1.

Our focus was on the rejection rate of the “4 out of 4” context, because that rejection generates the “some, but not all” pragmatic interpretation. The One-way Anova test for the condensed data showed a significant effect of the context [*F*_(1, 22)_ = 23.16, *p* < 8.3e-05]. Based on the data from Figure 1, we can infer that Kichwa participants did generate the “some, but not all” pragmatic reading. The percentage of rejection of the “4 out of 4” was 78.33% (47/60).

We found that Language Ability (measured on a scale of 1–5, the ability to speak, read, write, and listen in Kichwa or Spanish, where 1 = Kichwa, 5 = Spanish) positively correlates with Acceptance. Performing the simple linear model (*lm*) test, the Language Ability variable showed a significant impact on Acceptance, (*r*^2^ = 0.362, B = 1.033, SE = 0.434, *p* = 0.039). These results show that Spanish-leaning participants performed better in the task. Their higher bilingual ability gave them the advantage of performing better in this experimental task.

**Figure 2:**
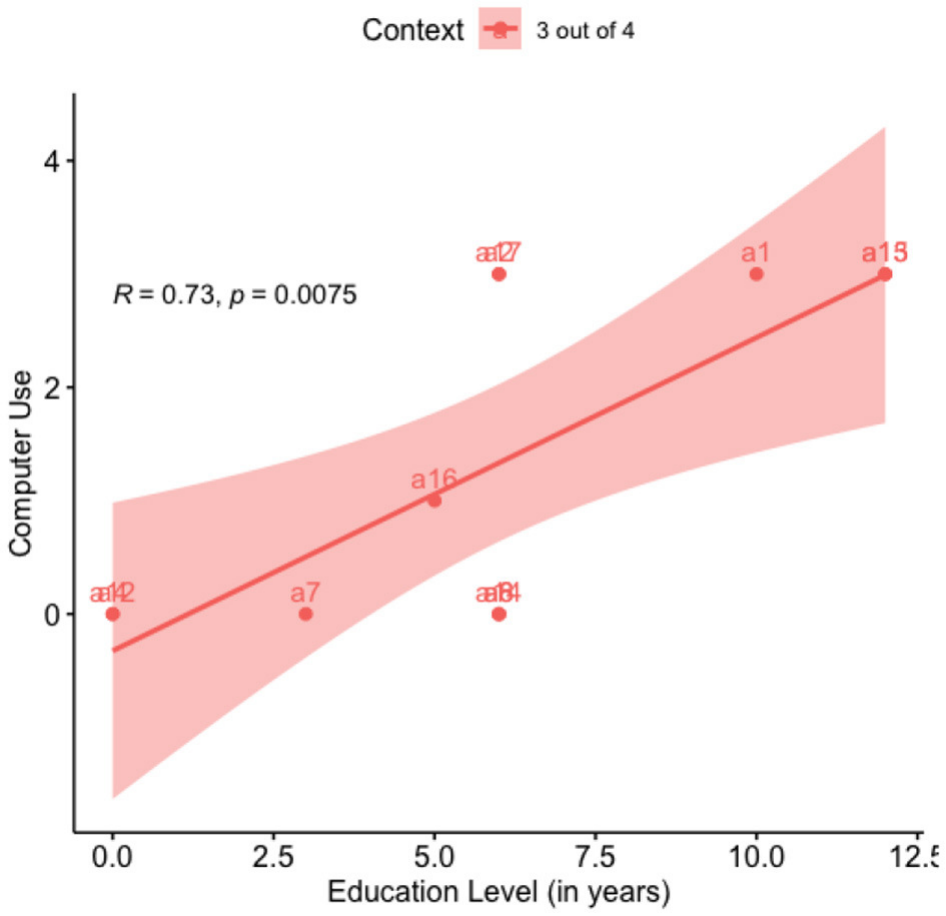
Regression: acceptance vs. education level.

As mentioned earlier in this section, Computer Use correlates positively with Education Years. Education Years significantly impact Computer Use, (*r*^2^ = 0.527, B = 1.91, SE = 0.571, *p* = 0.0075), which is expected as more educated individuals have more access to computers. This relationship is shown graphically in Figure 2.

### 2.5 Discussion experiment 1

Answering the first research question (i), our results showed that adult Kichwa speakers do not recognize two phonetic variants for the quantifier *wakin*, as we initially hypothesized. Even though we could show both versions of *wakin* successfully, speakers could not assign them different pragmatic readings. In that sense, the claim made by Faller and Hastings (2008, 308), where they state that “*wakin* is therefore more similar to English stressed *SOME* than the partitive some of (the)” seems to not apply to Imbabura Kichwa's *wakin*, at least insofar as it concerns phonetic form, and not meaning.

Our first research question (i) concerned the effect of the phonetic variant in pragmatic reading and the generation of the “some, but not all” interpretation. We were expecting that the final syllable stressed form [wa.ˈkiŋ] (in line with the first research question) would yield a “some, but not all” pragmatic reading more categorically than [ˈwa.kiŋ], similar to the English stressed form *SOME* (Grinstead et al., 2010; Thorward, 2009). That was not the case. There is only one form of the quantifier pronounced [ˈwa.kiŋ].

For our second research question (ii), adult Kichwa speakers could generate a “some, but not all” pragmatic interpretation in this experiment to some degree. Even though we got a 78.33% rejection of the “4 out of 4” context, these results are similar to the rates found for some of the experiments in other languages. (Janssens et al., 2014; Thorward, 2009; Vargas-Tokuda et al., 2009; Papafragou and Musolino, 2003; Noveck, 2001). Though, these results are not as categorical as in Pratt et al. (2018).

Our results also found that participants' years of education played a fundamental role in Acceptance rates. The inferential statistics showed a significant effect of Education in Acceptance for the “3 out of 4” context. Even more, we saw that the education variable strongly affected reaction time and Computer Use. As expected, the more educated individuals delivered the tasks faster than those with less education. Similarly, the more educated the individuals were, the more familiar they were with computers. We observe that this may be the cause as to why we did not get the expected highest acceptance rates in this experiment.

## 3 Experiment 2

The Kichwa results were not as categorical as expected. We considered this to be due to some intrinsic properties of the Kichwa quantifier *wakin*. To eliminate this probability of whether this is a Kichwa-intrinsic issue, we followed this up by testing Spanish monolingual and bilingual Kichwa-Spanish speakers' interpretation of the corresponding Spanish existential *algunos* to determine whether there might be some type of language contact distinction rooted in Kichwa causing the less categorical responses. That is, if there were some sort of general inclination rooted in Kichwa not to accept existential quantifiers in universal contexts, we might find an influence of it in the use of the analogous Spanish existential *algunos*.

1. Do Ecuadorean monolingual Spanish-speaking adults from different social backgrounds generate the “algunos, pero no todos = some, but not all” pragmatic implicature reading with the Spanish existential quantifier *algunos*?
2. Do Imbabura Kichwa-Spanish bilingual adults from different social backgrounds generate the “algunos, pero no todos = some, but not all” pragmatic implicature reading with the Spanish existential quantifier *algunos*?

### 3.1 Participants

This second experiment consisted of two different groups. A total of *N* = 20 adult Kichwa-Spanish bilinguals from rural Kichwa communities around Cotacachi and Otavalo participated in this experiment. They were different participants from the first experiment. Participants who did not pass the fillers (*N* = 5) were excluded from the data. A total of 15 participants (*M* = 29.8 years, SDev = 6.87 years, Range = 21–42 years) passed the fillers. Kichwa-Spanish bilingual participants in this experiment also filled out the Alberta Language Evaluation Questionnaire (ALEQ-3) by Soto-Corominas et al. (2020) to study whether sociolinguistic and extralinguistic factors are influencing or not the implicature reading in this population. These bilingual participants were also early sequential bilinguals as in our first experiment (Amengual, 2019, p. 956), they were exposed to Kichwa at birth and learned Spanish at school or later in life. The second group consisted of 20 adult Spanish monolingual speakers from Cotacachi, Imbabura. None of them were ethnically Kichwa individuals. A total of *N* = 18 participants passed the fillers (*M* = 31.50 years, SDev = 8.89 years, Range: 18–38 years). The monolingual participants of this experiment, even though they lived in Kichwa-speaking areas, did not speak a single word in Kichwa, nor had contact with other languages.

Table 6 shows the descriptive data for sociolinguistic variables from the ALEQ questionnaire for the Kichwa-Spanish bilingual participants.

**Table 6:** Sociolinguistic variables data.

**Category**	**Mean**	**Standard dev**.	**Range**
Age (in years)	29.80	6.87	21–42
Education years	8.67	4.62	0–12
Computer use	0.93	0.94	1–3
Internet access	1.87	2.03	1–3
Kichwa fluency	4.13	0.82	1–5
Language use	2.1	1.3	1–5
Language ability	2.59	0.68	1–5

### 3.2 Procedures

The stimuli discourse units used for this experiment were the Spanish version used in Mexico City by Grinstead et al. (2010). Similar to experiment 1, there were four warm-up sentences, four filler sentences and ten experimental sentences following the criteria of Question Under Discussion conversational discourse (Roberts, 2003). For the experimental stimuli, five (5) discourse sentences for *algunos* were created. They were used in both “3 out of 4” and “4 out of 4” contexts. By context, we mean that for each contexts, “3 out of 4” and “4 out of 4” cartoon characters are performing an action in a motion-animated audio and video scenario under the QUD paradigm.

### 3.3 Stimuli

For this experiment, we used a variation on the traditional TVJT, as first implemented by Grinstead et al. (2019). The audio content was adapted to the Ecuadorean Highland (Quiteño) Spanish variety. We recorded the audio directly into a personal computer via Audacity v.2.4.1. Recordings were made by the same Kichwa-Spanish-English trilingual female who recorded the Kichwa stimuli. No further changes were made. Here is one of the experimental stimuli examples.

(18) Los niño-s está-n en la casa. Quiere-n subir a ver la
tele.
The.PL child.PL Be-3PL in the house. Want-3PL climb to watch the television.
“The children are at home. They want to climb the ladder to go watch TV.”

Sentence (18) creates background information to set the stage for the upcoming scenario. The following sentence (19) gives more detailed information about the activity these cartoon characters are about to perform.

(19) Oh no! Las escalera-s son muy alta-s.
Uh-Oh the.PL ladder-PL be.PL very high.PL
“Uh-Oh! the ladder is too high.”

This is followed by the explicit Question Under Discussion in (20), which prepares participants to affirm or reject the final experimental sentence.

(20) Quién-es va-n a subir las escalera-s?
who-PL go-3PL to climb the.PL ladder-PL
“Who is going to climb the ladder?”

This sentence (21) contains our target quantifier, in which participants are expected to answer YES to the “3 out of 4” context and reject the “4 out of 4” context for all the experimental stimuli for the quantifier *algunos*.

(21) Ya se, *algunos* niño-s subi-eron las escalera-s
I know. some child-PL climb-PAST.PL the.PL ladder-PL
“Well, I know. *Some* children were able to climb the ladder”

The stimuli, including the warm-ups and fillers, were encoded in the Superlab v5 software. The four warm-up stimuli were placed at the beginning of the trials to familiarize participants with the upcoming experimental work. The experiment and filler stimuli were placed in random order. We did a within-subjects experimental design in which language was the distinctive factor.

Before starting the experiments, Kichwa-Spanish bilingual participants were asked to complete the ALEQ-3 (Soto-Corominas et al., 2020) questionnaire to capture the sociolinguistic information. Only the ALEQ results of the fifteen participants who passed the fillers are included for statistical purposes. Spanish monolingual participants were not asked to fill out the ALEQ-3 questionnaire. For the experimental part, participants were asked to press the C key on the keyboard, labeled with a happy face 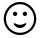, if they agree with the statement, or press the M key, labeled with a sad face 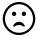, if they disagree with the statement.

### 3.4 Results

#### 3.4.1 Descriptive statistics

Table 7 shows the acceptance results for each context, “3 out of 4” and “4 out of 4” separated by each group or participants.

**Table 7:** Acceptance rate of *algunos* by language speakers.

**Context**	**Language speakers**			
	**Kichwa-Span-Bil**.	**%**	**Spanish Monol**.	**%**
3 out of 4	45/75	60%	79/90	87.78%
4 out of 4	33/75	44%	35/90	38.89%

We also added the Flanker Test to measure the inhibition component of executive function (EC) (Eriksen and Eriksen, 1974). In addition, we also measured the Reaction Time for both groups. See Table A1 for reference.

#### 3.4.2 Inferential statistics

First, we performed a two-by-two factorial ANOVA, having Group (Indigenous Kichwa-Spanish bilinguals and Mestizo Spanish monolingual participants) and Context (“3 out of 4” and “4 out of 4”) as our two categorical variables and Acceptance as our dependent variable. We found a significant effect of the context in the Acceptance [*F*_(1, 62)_ = 11.71, *p* > 0.05] as well as no significant effect of the group by context interaction [*F*_(1, 62)_ = 2.726, *p* > 0.05].

**Figure 3:**
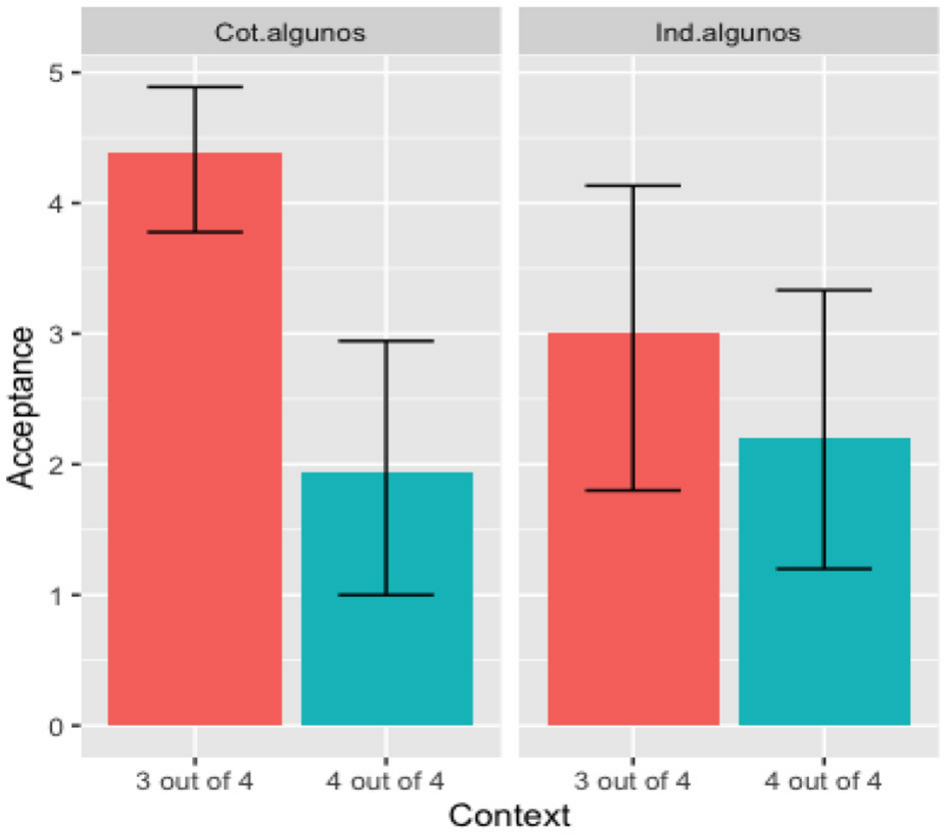
Mean acceptance by language group (*algunos*).

Though there was a significant effect of Context and no significant effect of Group, Figure 3 suggests that monolingual Spanish speakers, not the bilingual participants, drive the main effect of Context. A follow-up *t*-test shows a significant difference in rejecting the “4 out of 4” contexts by monolinguals [*t*_(34)_ = 4.05, *p* < 0.001] but not by bilinguals [*t*_(28)_ = 0.978, *p* > 0.05].

We wanted to know what is causing these low acceptance rates for the Kichwa-Spanish bilinguals in the “3 out of 4” context and the low rejection rate for bilinguals and the Spanish monolingual speakers for the “4 out of 4” context. We performed the Pearson correlation (two-tailed) to observe if some of the ALEQ-3 sociolinguistic variables predicted tendencies on the acceptance rate for each of the contexts for bilingual speakers. For the “4 out of 4” context that delivers the “some, but not all” interpretation, we find the correlation values in Table 8.

**Table 8:** SES correlation for the “4 out of 4” context.

	**Accep**.	**Age**	**Educ.Y**	**Comp.Use**	**Lang.Use**
Accep	1	0.30	**−0.53**	−0.24	−0.33
Age		1	−0.35	**−0.55**	−0.17
Educ.Y			1	**0.71**	0.20
Comp.U				1	0.09
Lang.Use					1

Bold values indicate that these variables correlated with Acceptance in the 4 out of 4 context.

The correlation table show us that there is a moderate to strong negative correlation of Educ.Years with Acceptance (two-tailed), (*r* = −0.53, *p* < 0.001). We also found that there is a strong positive correlation between Educ.Years and Computer Use (two tailed), (*r* = 0.71, *p* < 0.001.) Also, Age of participants correlates negatively with Computer Use (two tailed), (*r* = −0.55, *p* < 0.001).

**Figure 4:**
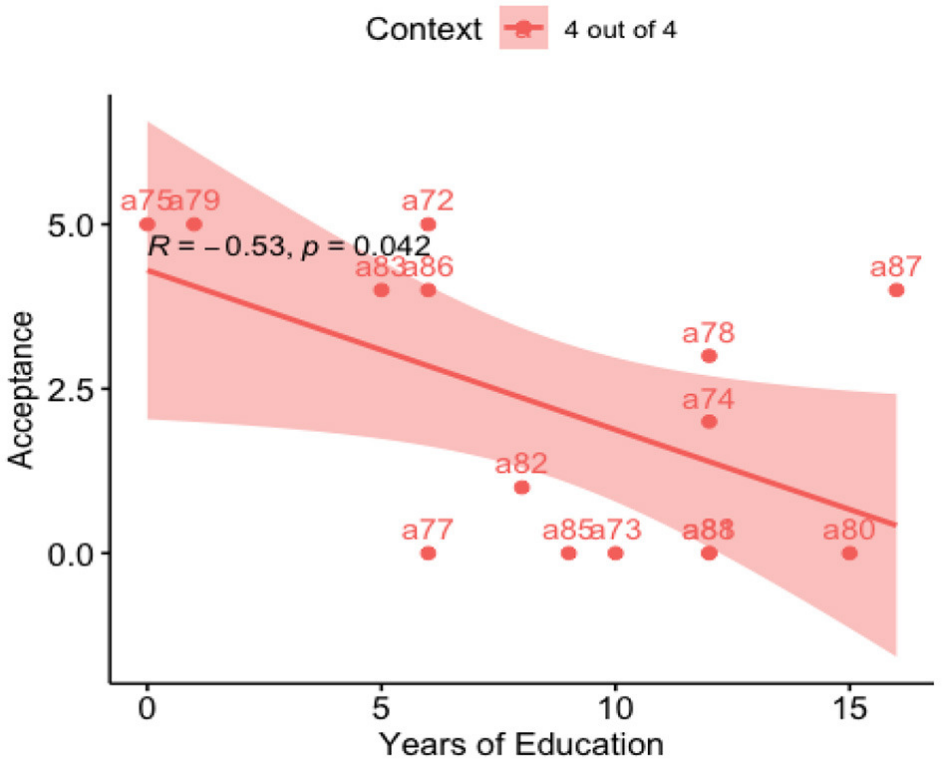
Regression line: acceptance vs. education years.

Performing the simple linear regression for Acceptance rate in function of Education Years for participants, we found that Education Years explains 28.24% of the Acceptance outcome, (*R*^2^ = 0.282, β = −0.24, SE = 0.11, *p* = 0.04) (see Figure 4).

For the “3 out of 4” context, we also performed the Pearson two-tailed correlation tests, as seen in Table 9.

**Table 9:** SES correlation for the “3 out of 4” context.

	**Accep**.	**Age**	**Educ.Y**	**Comp.Use**	
Accep	1	−0.47^.^	**0.70**	**−0.67**	−0.33
Age		1	−0.35	**−0.55**	−0.17
Educ.Y			1	**0.71**	0.20
Comp.U				1	0.09
Lang.Use					1

Bold values indicate that these variables correlated with Acceptance in the 3 out of 4 context.

We found that for the “3 out of 4” contexts, Education Years correlate strongly with the Acceptance rate (two-tailed), (*r* = 0.70, *p* = 0.003). We also found a strong negative correlation between Computer Use and Acceptance rate (two-tailed), (*r* = 0.67, *p* = 0.006).

**Figure 5:**
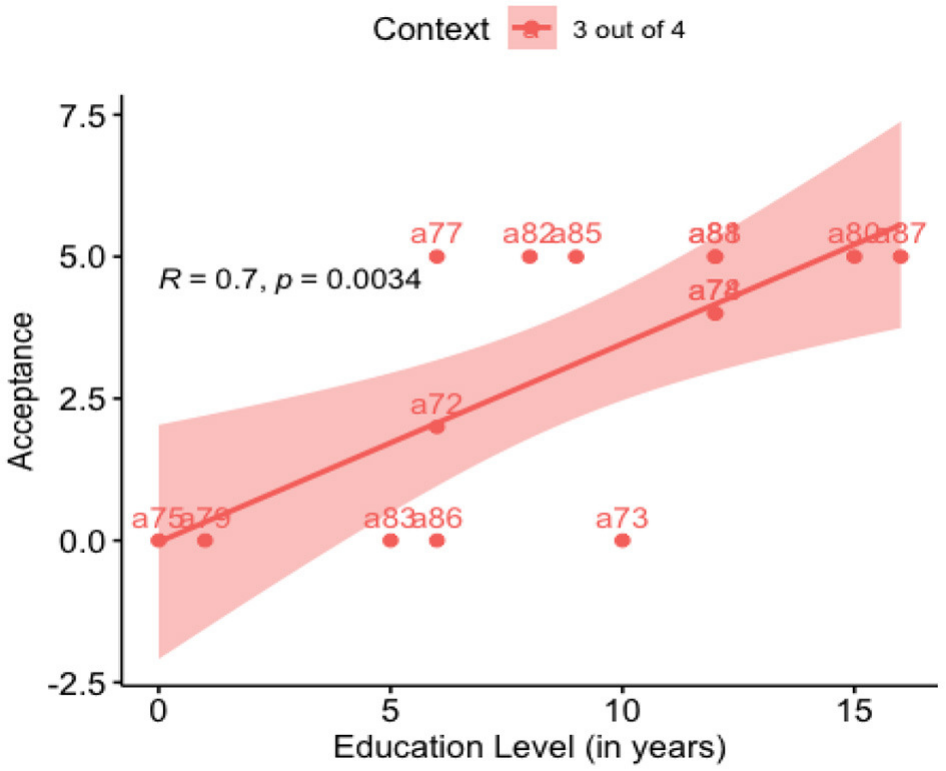
Regression line: acceptance vs. education years.

**Figure 6:**
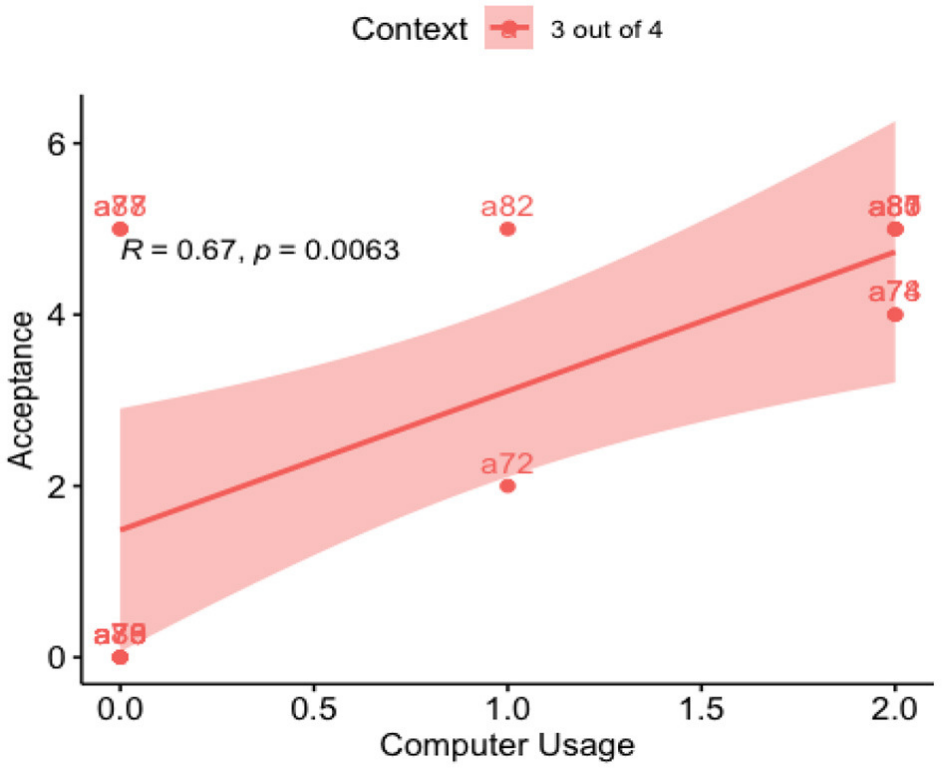
Regression line: acceptance vs. computer use.

Performing a simple linear regression (lm) of years of education on Acceptance rate in 3 out of 4 contexts, we found that Years of Education explains 49.61% of the Acceptance outcome, (*R*^2^ = 0.496, β = 0.35, SE = 0.096, *p* = 0.003). We also found that Computer Use explains 44.87% of the Acceptance rates, (*R*^2^ = 0.4487, β = 1.62, SE = 0.499, *p* = 0.006) (see Figures 5, 6).

**Figure 7:**
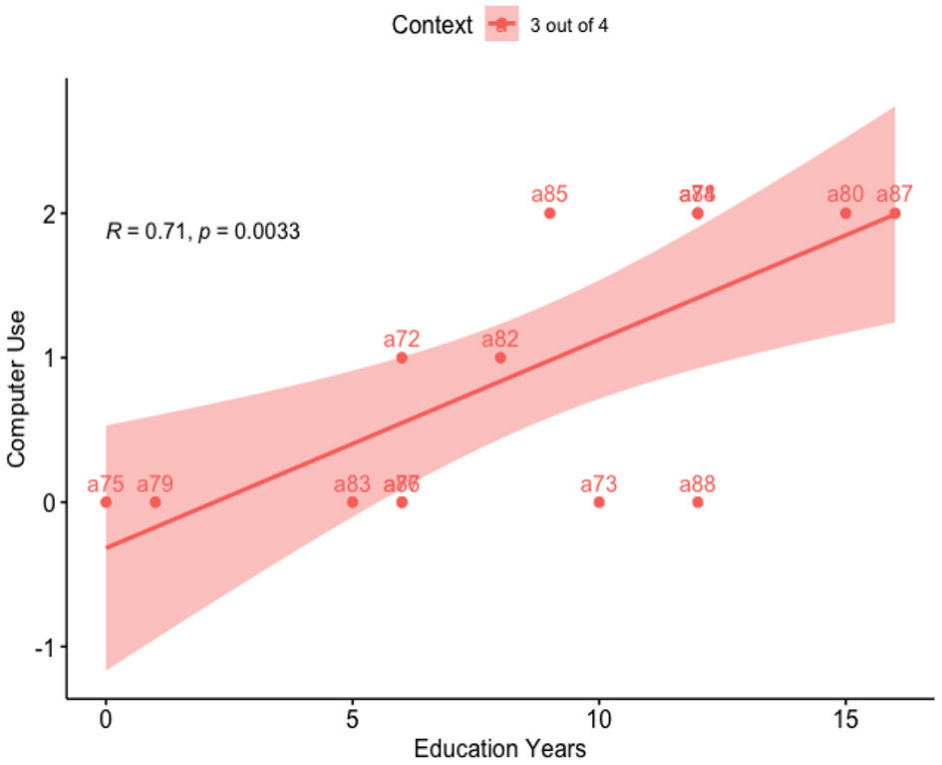
Regression line: computer use vs. education years.

So far, we have seen that Education Years strongly predict the Acceptance rate in both contexts. Also, we have seen that Computer Use predicted the Acceptance rate for the “3 out of 4” context. Measuring Computer Use in function of Education Years we found that Education Years explains 49.9% of Computer Use levels, (*R*^2^ = 0.499, β = 3.45, SE = 0.96, *p* = 0.003) (see Figure 7).

#### 3.4.3 The cognitive factor

Concerning the inhibition component of Executive Function, measured here by the Flanker Task, monolinguals have significantly higher inhibition than bilinguals [*t*_(64)_ = 4.9338, *p* < 0.001]. Further, regarding language processing, monolinguals were significantly faster than bilinguals, [*t*(64) = −5.812, *p* < 0.001). However, neither of these factors was predictive of acceptance, in either group, in either context, *p* > 0.05.

### 3.5 Discussion experiment 2

First, the Spanish monolingual participants interpreted the “some, but not all” pragmatic implicature with the Spanish quantifier *algunos*. However, this pragmatic interpretation was not as high as expected, [as in Pratt et al. (2018)] partially answering the third research question of delivering pragmatic implicature with the Spanish quantifier algunos. Their results are lower than the results from experiment 1 with the Kichwa quantifier *wakin*.

Second, regarding the Kichwa-Spanish bilinguals, we saw that they could not generate the “some, but not all” interpretation with the Spanish quantifier *algunos* due to the considerable variation in acceptance rates.

The fourth research question was answered negatively by these participants. Kichwa-Spanish bilinguals could not reject the *4 out of 4* context and differentiate it from the “3 out of 4” context. They were also not able to judge categorically as true the “3 out of 4” context, which was expected to be accepted at high rates.

Interested in what may be affecting them in delivering the expected reading, the correlation table showed us that two main variables affected the outcome: Participants' years of Education and Computer Use abilities. The Kichwa-Spanish bilinguals sample for experiment 2 were not highly educated (*M* = 8.67 years of education). Comparatively, as they are not highly educated, they do not have access to technology such as computers and are not familiar with their usage. We think that this is the crucial finding from experiment 2. The results suggest that this non-familiarity with Computer Use strongly impacted our results for experiment 2. Participants familiarity with the technological equipment used in experiments is a critical factor to consider when planning linguistic experimental designs.

For our point of interest, Figure 4 shows a fascinating negative correlation, where participants who have more years of education judge as false the “4 out of 4” context more than those with fewer years of education. The same was true for the “3 out of 4” context, which participants were expected to judge as true. We also looked at the linguistic variables from ALEQ-3, such as Language Ability, Kichwa Fluency, or Language Use; none of these variables impacted the results, as we saw in the correlation Tables 8, 9.

No linguistic background tests similar to ALEQ-3 were conducted for the Spanish monolingual population. However, by simple observation, these participants' sociolinguistic status and access to technological resources were similar to those of the Kichwa-Spanish bilinguals. They share similar limitations in access to resources and technology in the geographical region where they were tested.

Analyzing the Flanker test, we found that monolingual speakers had higher inhibition scores for the Executive Function (EF) inhibition component. However, those more outstanding access scores did not give them any cognitive advantage over our bilingual participants in Acceptance rates in any of the contexts studied for Reaction Time. We also found that Spanish monolinguals processed the stimuli faster than their bilingual counterparts. In the same trend as the linguistic inhibition test, language processing time did not give monolinguals any advantage in any of the contexts studied.

We assume, then, that if none of the linguistic factors (tested language, Language Dominance, Language Ability, Fluency) are conditioning the outcome of the results, we look for answers elsewhere. We saw that access to computers, the ability to use them, and years of education were crucial factors that conditioned the outcome. This key factor may have also affected the Flanker test. We needed to change how we approached the experiment design and the materials we used on it. We understood that pushing buttons on a computer keyboard to accept or reject the TVJT sentences was an obstacle for the bilingual participants. We concluded then that we would not use the Superlab software for future experiments.

## 4 Experiment 3

In the preceding experiment, it was contemplated that our methodological strategy might not be suitable for our target population. Consequently, we opted to modify the methodological approach for the subsequent experiments involving our participants. We accommodated our strategies and methodologies to make it more suitable and (culturally) appropriate for our target population as suggested by Sanchez (2006). We modified the answer form to simply ask participants to say *CIERTO = True* or *FALSO = False* according to their judgment of each experimental stimulus presented in the study. Alterations were also made in the criteria for participant selection; we chose university students from Quito and Guayaquil. These participants are exclusively monolingual speakers of Quiteño Spanish and Guayaquil (Costeño) Spanish. In this current experiment, our objective is to address the identical research question (iii) posed in the previous experiment.

1. Do Ecuadorean monolingual Spanish-speaking adults from different social backgrounds generate the “algunos, pero no todos = some, but not all” pragmatic implicature reading with the Spanish existential quantifier *algunos*?

### 4.1 Participants

For this third experiment, two different groups of participants were selected in Quito and Guayaquil. In Quito, a total of *N* = 18 university students, all adult Spanish monolinguals (*M* = 22.89 years, SDev = 4.98 years, Range: 18–34 years), were selected. In Guayaquil, a total of *N* = 15 university students, all adult monolingual Spanish speakers (M = 21.20 years, SDev = 2.73 years, Range = 18–29 years), were selected. None of these participants failed the filler stimuli. The geographical spaces where these participants were selected do not have linguistic influence from Kichwa or any other languages. Their surrounding linguistic spaces were entirely Spanish.

### 4.2 Procedure

In light of the results from Experiment 2, we opted against utilizing the Superlab software on the computer for these participants. The same discourse stimuli as in Experiment 2 were employed. These discourse units underwent editing via iMovie 10.1.11 in the following manner:

1. The four warm-up videos were compressed together, with 5 s between each discourse. They were shown to participants using the researcher's personal computer. After each warm-up stimulus, the video was paused to explain to them what was expected of them to do for the upcoming experimental videos. After completing the warm-up units, participants were told to answer by judging the statements as True or False according to their judgment.
2. Subsequently, three distinct versions of experimental videos (including fillers) were created. These were arranged in a random sequence. Each experimental video segment was interspersed with a 5-s pause to afford participants sufficient time to transition from one segment to the subsequent one.
3. In addition, the Flanker Test was administered to the participants as part of the research methodology.

In the current experiment, the Superlab software was not utilized, hence no measurements of Reaction Time (RT) were recorded. Furthermore, the administration of the ALEQ-3 questionnaire was not conducted for the participants involved in the third experiment.

### 4.3 Results

#### 4.3.1 Descriptive statistics

Here in Table 10 we have the Acceptance vs. Context results for participants from Quito and Guayaquil.

**Table 10:** Acceptance rate of *algunos* by cities.

**Context**	**Cities**			
	**Quito Monol**.	**%**	**Guayaquil Monol**.	**%**
3 out of 4	86/90	95.56%	73/75	97.33%
4 out of 4	3/90	3.33%	5/75	6.67%

The Flanker Test results for Quito and Guayaquil participants are given in Table A2.

#### 4.3.2 Inferential statistics

Initially, a two-by-two factorial ANOVA was conducted with Group (participants from Quito and Guayaquil) and Context (“3 out of 4” and “4 out of 4”) as the categorical independent variables, and Acceptance as the dependent variable. The analysis revealed a significant main effect for Context on Acceptance rates, [*F*_(1, 62)_ = 1146.8, *p* < 0.001]. However, no significant main effect was observed for Group (Quito vs. Guayaquil) on Acceptance, [*F*_(1, 62)_ = 0.3, *p* > 0.05] nor was there a significant interaction between Group and Context on Acceptance rates, [*F*_(1, 62)_ = 0.0, *p* > 0.05].

**Figure 8:**
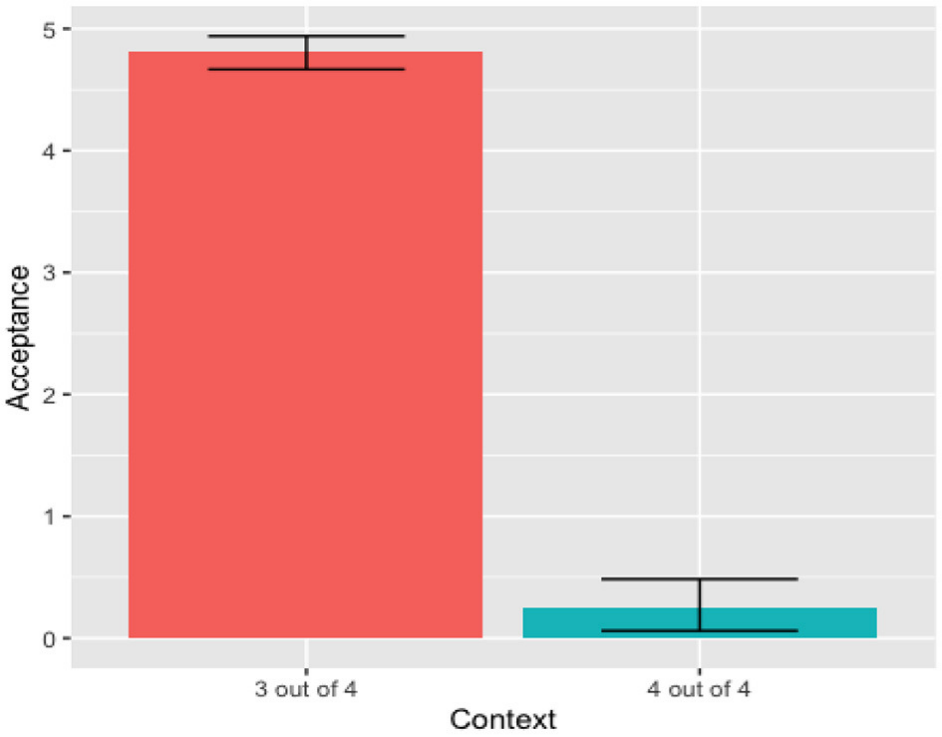
Regression line: computer use vs. education years.

Interested in exploring if there was a difference in the Acceptance rates between participants from Quito and Guayaquil within each context, an independent samples *t*-test was performed for both contexts. In the “3 out of 4” context, no significant difference in Acceptance rates between Quito and Guayaquil participants was found, [*t*_(31)_ = 0.64, *p* > 0.05], similar to findings in the “4 out of 4” context, [*t*_(31)_ = 0.714, *p* > 0.05]. Given the absence of mean differences, data consolidation into a single dataset (see Figure 8).

The results indicate that participants from Quito and Guayaquil successfully rejected the “4 out of 4” context 95.15% of the time (8/165, see Table 10) and deliver the “some, but not all” implicature interpretation with the Spanish quantifier *algunos*. The Flanker test for measuring executive function did not exhibit any predictive capacity regarding the Acceptance outcome across various contexts, *p* > 0.05.

### 4.4 Discussion experiment 3

The third research inquiry was conclusively addressed, affirming that Ecuadorean Spanish-speaking monolinguals can deliver the “algunos, pero no todos = some, but not all” pragmatic interpretation with the Spanish quantifier *algunos*. Quito and Guayaquil participants were able to deliver the expected pragmatic interpretation categorically. The results obtained were higher than any of the tests delivered in other languages, Noveck (2001); Papafragou and Musolino (2003); Thorward (2009); Vargas-Tokuda et al. (2009); Janssens et al. (2014), and similar to Pratt et al. (2018). The rejection rate for the “4 out of 4” context was 95.15%.

By opting for a simple True or False judgment of the stimuli instead of using the computer keyboard for Acceptance under the Superlab software, we achieved a strong rejection rate for the “4 out of 4” context and a very similar rate of acceptance for the “3 out of 4” context. Concurrently, the participants in this experiment were more educated and younger compared to those in experiment 2. Although we lack a direct measure of these participants' familiarity with computers, their significantly higher levels of education compared to the participants in experiment 2 are noteworthy.

## 5 Experiment 4

The outcomes of our prior research have prompted us to contemplate conducting a fourth experiment. This experiment would involve Kichwa-Spanish bilinguals who have received a high level of education and would focus on the use of the Kichwa quantifier *wakin*. We tentatively attribute the categorical judgments of the stimuli in this demographic to the absence of methodological barriers that could potentially hinder the measurement of their semantic-pragmatic knowledge.

As a result, we were inclined to revisit the second research question, albeit with minor modifications, by employing only one variant of the quantifier *wakin*. This decision is informed by the findings from our initial experiment, which revealed that Kichwa participants were unable to distinguish between two different phonetic forms of *wakin*. Our third experiment also demonstrated that Spanish monolingual participants with a high level of education categorically interpreted the “algunos, pero no todos = some, but not all” implicature reading (95%). For the fourth experiment, we have chosen participants whose educational backgrounds are similar to those in experiment 3. In this fourth experiment, we aim to answer the following research questions:

1. Do Imbabura Kichwa-Spanish bilingual adults from different social backgrounds generate the “wakin, mana tukuy = some, but not all” pragmatic implicature reading with the Kichwa existential quantifier wakin?
2. Do participants' sociolinguistic variables influence their pragmatic interpretation of the existential quantifier *wakin* in Kichwa?

### 5.1 Participants

For this fourth experiment, *N* = 30 highly-educated (in Spanish) Kichwa-Spanish bilingual participants were selected (*M* = 25.1 years, SDev = 4.17 years, Range: 18–32 years). They were all from around the city of Cotacachi. All of them have spoken Kichwa since birth. None of these participants failed the filler stimuli. These bilingual participants, similar to the other bilingual groups, were early sequential bilinguals Amengual (2019 p. 956), meaning that their birth language is Kichwa and learned Spanish at school or later in life.

Table 11 presents the descriptive statistics for sociolinguistic variables from the ALEQ questionnaire for the Kichwa-Spanish bilingual participants.

**Table 11:** ALEQ results for high Ed. bilinguals.

**Category**	**Mean**	**Standard dev**.	**Range**
Age (in years)	25.1	4.17	18–32
Education years	14.25	2.26	12–18
Computer use	3	0.0	1–3
Internet access	2.43	0.704	1–3
Kichwa fluency	3.85	0.92	1–5
Language use	2.35	0.67	1–5
Language ability	2.13	0.45	1–5

### 5.2 Procedure

Informed by the insights gleaned from Experiments 2 and 3, we opted not to employ the Superlab software for the participants in the current study. Instead, we utilized the stimulus discourse that was developed for the unstressed variant of *wakin* in Experiment 1. These discourse segments were subsequently edited using iMovie version 10.2.1, adhering to the identical procedure employed in Experiment 3.

1. Initially, the researcher introduced a series of four warm-up videos via a personal computer. Following each warm-up stimulus, a pause was implemented to clarify the anticipated responses from the participants for the upcoming experimental videos.
2. The sequence of the experimental and filler videos was randomized, ensuring an equitable distribution of participants across each sequence. Participants were instructed to evaluate the truthfulness of the statements as either “True” or “False” based on their discernment.

For the bilingual participants involved in this fourth experiment, the ALEQ-3 questionnaire was administered, and the Flanker test was also conducted.

### 5.3 Results

#### 5.3.1 Descriptive statistics

Table 12 shows the Acceptance vs. Context results for the Kichwa-Spanish bilingual speakers with higher levels of education.

**Table 12:** Acceptance rate for high Ed. bilinguals.

**Context**	**Acceptance**	**Rate %**
3 out of 4	99/100	99%
4 out of 4	3/100	3%

Table A3 shows the Flanker test results for the Kichwa-Spanish highly educated participants.

#### 5.3.2 Inferential statistics

An independent two-sample *t*-test was conducted to evaluate the mean Acceptance values for both the “3 out of 4” and “4 out of 4” contexts. The *t*-test unveiled significant disparities between the contexts, [*t*_(38)_ = 39.9, *p* < 0.0001]. In the “4 out of 4” context, participants in this fourth experiment rejected experimental sentences as false 97% (97/100) of the time, demonstrating their capacity to deliver a categorical implicature interpretation of “wakin, mana tukuy = some, but not all” with the Kichwa existential quantifier *wakin*. Additionally, a two-tailed Pearson correlation was computed in *R* for each of the contexts under investigation against the sociolinguistic variables derived from the ALEQ-3 questionnaire. For detailed results, refer to Table 13 below.

**Table 13:** SES correlation for the “3 out of 4” context.

	**Accep**.	**Age**	**Educ.Y**	**Comp.Use**	**Lang.Use**	**Flanker**
Accep	1	0.228	0.231	NA	0.121	0.072
Age		1	0.399	NA	0.255	0.172
Educ.Y			1	NA	0.413	−0.021
Comp.U				1	NA	NA
Lang.Use					1	−0.242

In the fourth experiment involving highly educated Kichwa-Spanish bilinguals, no sociolinguistic variables were found to predict the Acceptance rate within the “3 out of 4” context. The correlation Table 14 below, pertains to the “4 out of 4” context.

**Table 14:** SES correlation for the “4 out of 4” context.

	**Accep**.	**Age**	**Educ.Y**	**Comp.Use**	**Lang.Use**	**Flanker**
Accep	1	−0.059	−0.316	NA	.044	−0.112
Age		1	.0.399	NA	0.255	0.171
Educ.Y			1	NA	0.413	−0.021
Comp.U				1	NA	NA
Lang.Use					1	−0.242

**Figure 9:**
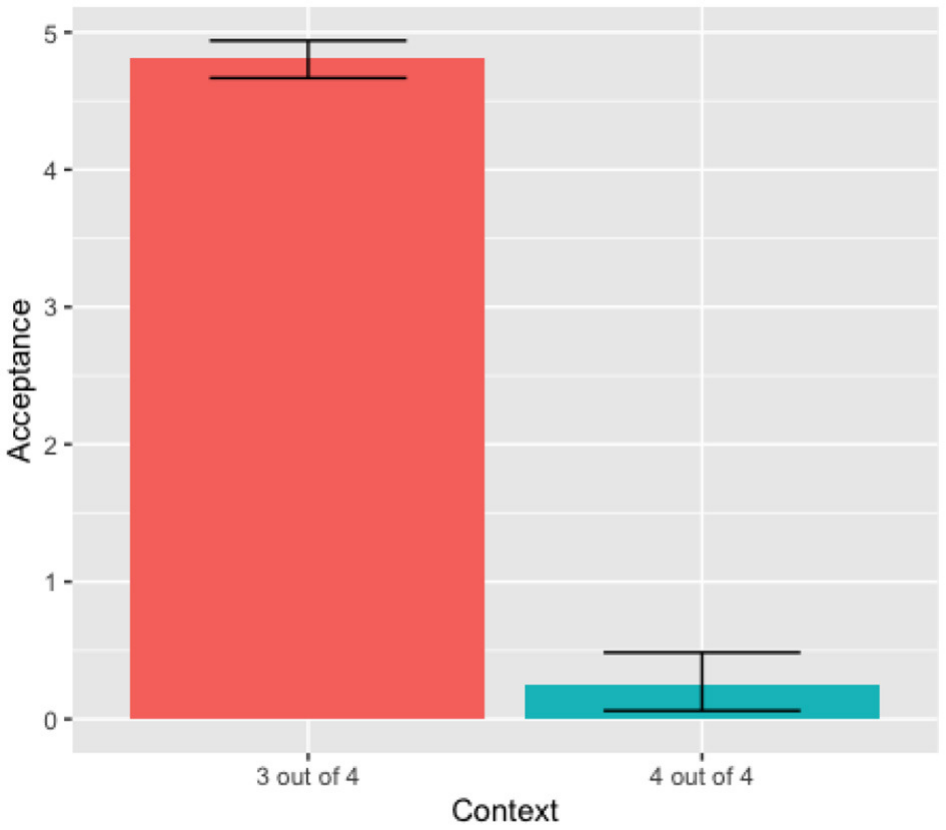
Mean acceptance by Kichwa-Spanish bilinguals (*wakin*).

In the fourth experiment, it is observed that none of the sociolinguistic variables are predictive of the Acceptance rate for the “4 out of 4” context among highly educated Kichwa-Spanish bilinguals. Moreover, when considering the categorical acceptance rates from the same experiment, neither the scores from the ALEQ-3 questionnaire's sociolinguistic variables nor the Flaker test scores exhibited any influence on the acceptance outcomes (see Figure 9).

### 5.4 Discussion experiment 4

The results from the fourth experiment affirmatively address the fourth research question, which asks, *Do Imbabura Kichwa-Spanish bilingual participants generate the “wakin, mana tukuy = some, but not all” pragmatic interpretation with the Kichwa existential quantifier wakin?* It has been established that Kichwa speakers can indeed generate the “some, but not all” interpretation with the Kichwa quantifier *wakin*.

The outcomes from the fourth experiment provide a tentative affirmative response to the fifth (v) research question, which pertains to the influence of various sociolinguistic variables on the pragmatic interpretation of quantifiers in Imbabura Kichwa. The participants in this experiment were significantly more educated compared to those in experiment 1. Furthermore, unlike in experiment 1 where participants utilized a keyboard pressing methodology within the Superlab software, participants in experiment 4 responded to prompts with a straightforward True or False. In experiment 1, the “4 out of 4” context was rejected as false 78.33% of the time, whereas in experiment 4, this rejection rate increased to 97%. We consider that participants' technology management played a crucial role in obtaining these results.

## 6 General discussions

### 6.1 The Kichwa quantifier *wakin* (condensed)

A comparative analysis of the mean scores for the ALEQ-3 results, derived from two distinct sample groups that participated in the experiments on the Kichwa existential quantifier *wakin*, is crucial. Comprehending the sociolinguistic commonalities and differences between these groups can yield vital insights that enhance the understanding of the experimental outcomes. A two-tailed independent sample *t*-test was utilized to compare the mean values of the relevant ALEQ-3 variables employed in experiments 1 and 4, as seen in Table 15.

**Table 15:** *t*-test values Experiment 1 vs. Experiment 4.

**Category**	**Means Exps**		**Test values**		
	**Exp. 1**	**Exp. 4**	***t*-test**	**df**	***p*-value**
Age (in years)	35.53	25.1	4.4994	62	**< 0.001**
Education years	6.0	14.25	−10.832	62	**< 0.001**
Computer use	1.33	2.0	−2.8945	62	**0.005**
Internet access	1.125	4.1	−10.474	62	**< 0.001**
Kichwa fluency	4.83	3.85	4.499	62	**< 0.001**
Language use	1.389	2.35	−4.642	62	**< 0.001**
Language ability	2.43	2.13	4.4994	62	0.08

Bold values indicate that the sociolinguistic categories tested in experiments 4 and 1 significantly differed statistically.

The data presented in Table 15 elucidates the distinct characteristics of the two groups involved in the experiments. Participants in experiment 1 were significantly older and had a lower level of education compared to those in experiment 4, *p* = < 0.001 as seen Table 15. These sociolinguistic variables also indicate that participants in experiment 1 had less familiarity with Computer Use, *p* = 0.05 and, overall, less internet access (*p* = < 0.001) than participants in experiment 4. These variables are interconnected, as lower education levels correlate with reduced access to technological resources such as computers and the internet.

From a linguistic perspective, participants in experiment 1 demonstrated greater fluency in Kichwa and used Kichwa more frequently in their daily interactions than participants in experiment 4, *p* < 0.001. In terms of language abilities (reading, writing, listening, and understanding), both groups exhibited similar capabilities, with no significant differences, *p* > 0.05. However, participants from experiment 1 were more Kichwa-dominant, while participants in experiment 4 were balanced bilinguals, utilizing both Spanish and Kichwa equally in their daily communication with their closest peers.

Upon comparing our ALEQ variables to the Acceptance results, it was observed that participants from experiment 1 rejected the “4 out of 4” context (the context that bears the “some, but not all” implicature interpretation) 78.33% (13/60) of the time. Meanwhile, participants in experiment 4, rejected the “4 out of 4” context in 97% (97/100) of the time. The independent two-sample *t*-test shows that this difference in Acceptance for the “4 out of 4” context is significant, [*t*_(30)_ = 2.42, *p* = 0.022].

For the Kichwa existential quantifier *wakin*, a step-wise multiple linear regression model (lm) (Venables and Ripley, 2002; Achim and Torsten, 2002) was implemented with all the ALEQ variables, as outlined in Table 15. Data from experiments 1 and 4 were combined into a single group for this calculation. We did this calculation for each context studied.

For the Kichwa existential quantifier *wakin*, a step-wise multiple linear regression model (lm) was implemented with all the ALEQ variables, as outlined in Table 15. Both, experiment 1 and experiment 4 data were condensed into one group for this calculation. We did this calculation for each context.

For the “3 out of 4” context, the best step-wise fit model was the model that included Education Years and Language Ability as independent variables and Acceptance as the dependent variable as seen in Table 16.

**Table 16:** Regression of the “3 out of 4” contexts by education years and language ability.

**Coefficients**	**Estimate**	**Std. error**	***t*-value**	***p*-value**
(Intercept)	2.38	0.594	4.01	< 0.001
Educ.years	0.098	0.03	3.260	0.003
Language ability	0.542	0.22	2.44	0.02

A significant regression equation was identified [*F*_(2, 29)_ = 9.082, *p* < 0.001], yielding an *R*^2^ value of 0.39. Among the variables studied, years of education and Language Ability emerged as significant predictors for the Acceptance rate of the “3 out of 4” context, as delineated in Table 16. Participants with a higher level of education from experiment 4 exhibited a greater acceptance rate of the “3 out of 4” context as true was higher compared to the less educated group from experiment 1. Other variables from the ALEQ-3 questionnaire, as seen in Table 16, did not significantly influence the Acceptance rate for the Kichwa quantifier *wakin*.

In the “4 out of 4” context, the optimal step-wise fit model incorporated years of education as an independent variable and acceptance as the dependent variable, as detailed in Table 17.

**Table 17:** Regression results of the “4 out of 4” context by age and education years.

**Coefficients**	**Estimate**	**Std. error**	***t*-value**	***p*-value**
(Intercept)	2.811	0.857	3.282	**0.003**
Age	−0.026	0.019	−1.390	0.175
Educ. years	−0.139	0.039	−3.546	**0.001**

Bold values indicate that the sociolinguistic categories tested in experiments 4 and 1 significantly differed statistically.

A significant regression equation was found [*F*_(2, 29)_ = 6.307, *p* = 0.005], with an *R*^2^ = 0.303. Among the variables studied, only years of education proved to be a significant predictor for Acceptance (β = −0.139, SE = 0.019, *p* = 0.0014). Other variables from Table 15 did not significantly impact the overall Acceptance rate for both contexts examined.

Our findings indicate that Kichwa speakers, on the whole, can discern the differences between the “3 out of 4” and the “4 out of 4” contexts. By rejecting the “4 out of 4” context (where 4 animated characters are performing an action), they can interpret the “some, but not all” implicature reading with the Kichwa indefinite quantifier *wakin*. However, these acceptance rates varied significantly among each group due to the methodological approach employed and the participants' sociolinguistic differences.

### 6.2 Kichwa *wakin* vs. Spanish *algunos*

Our observations indicate that individuals with a high level of education categorically rejected the “4 out of 4” context while consistently accepting the “3 out of 4” context in both Spanish and Kichwa. In the case of Spanish *algunos*, there were 33 highly educated participants from Quito and Guayaquil (see Table 10), and for Kichwa *wakin*, there were twenty 20 highly educated participants (see Table 12).

An independent *t*-test was conducted to determine if the sample means for both scenarios were statistically indistinguishable. The results indicated no significant difference in acceptance rates for *algunos* (*M* = 4.82) compared to Kichwa's *wakin* (*M* = 4.95) for the “3 out of 4” context, [*t*_(51)_ = −1.3724, *p* > 0.05]. Similarly, a two-sample *t*-test revealed no significant difference in Acceptance rates for *algunos* (*M* = 0.24) vs. Kichwa's *wakin* (*M* = 0.15) for the “4 out of 4” context, [*t*_(51)_ = 0.5399, *p* > 0.05].

The highly educated respondents demonstrated a clear distinction between scenarios across both languages, with their responses showing no significant variance. Their near-unanimous rejection of the “4 out of 4” context and their interpretation aligning with the “some, but not all” pragmatic understanding were consistent with the findings from Mexico City reported by Pratt et al. (2018).

## 7 Conclusions

The experimental methodology employed to investigate whether indigenous languages can convey pragmatic interpretations using their existential quantifiers yielded positive outcomes. Specifically, in the Kichwa language, no phonetic variations in their quantifiers were detected, contrary to the anticipated native speaker bias. Indigenous participants were able to consistently deliver the “wakin, mana tukuy = some, but not all” pragmatic implicature interpretation in their language considering sociolinguistic factors such as technological proficiency and educational attainment.

Similarly, for the Spanish quantifier *algunos*, participants demonstrated their ability to deliver the “algunos, pero no todos = some but not all” pragmatic implicature interpretation categorically in their language considering the same sociolinguistic variables as Kichwa-Spanish bilinguals do in Kichwa.

In both linguistic contexts and particularly within Kichwa, it was discerned that Education—interpreted as access to formal education—was a significant predictor of implicature comprehension. This aligns with broader linguistic research indicating that educational access enhances overall linguistic knowledge.

In the geographical area where this experiment was conducted, access to formal education in Kichwa is nonexistent. The entire education system is administered in Spanish and supported by Eurocentric, urban, monolingual ideologies. Under this system, Indigenous people are still associated with rurality and the education system in rural communities. Our participants in experiment 1 had limited access to formal education, which appeared to significantly influence the results. Rural or small city areas have deficient access to formal education, which is reflected in their abilities in language interpretation. However, a more exhaustive study of this variable and other pragmatic studies beyond quantifiers will be required to conclusively claim that education level is a crucial factor in delivering pragmatic interpretations in Kichwa.

A significant limitation of this investigation was the challenge associated with finding monolingual Kichwa participants. Within the research area, bilingualism in Kichwa and Spanish is prevalent, even among the older demographic, with varying degrees of proficiency in both languages. The participant count, particularly in the first experiment, was also limited due to particularly being an exploratory condition of this first experimental design. Consequently, the findings may not wholly represent the broader demographic segment. However, the results of subsequent experiments lend greater credence to these initial findings.

This pioneering research into the pragmatic interpretation of quantifiers in indigenous languages offers valuable perspectives on experimental methodologies while underscoring the importance of comprehending the community, social structure, and the use of technology when conducting research with similar demographic groups (Sanchez, 2006). Despite its limitations, the study makes a significant contribution to the understanding of language interpretation in indigenous communities. It also highlights the importance of considering sociolinguistic variables in such research. Moreover, this study breaks new ground in the experimental research into the pragmatic interpretation of quantifiers in an indigenous language like Kichwa.

Working in rural indigenous communities, the use of technology can give advantages to the researcher in terms of time and data processing. However, it can be counter-productive if participants are unfamiliar with technology or have no access to it. Despite technological issues faced during our first two experiments, our participants could categorically deliver the expected “some, but not all” pragmatic implicature. Further studies on the pragmatic interpretation and language production of adults and children in this and other Amerindian languages can build on the experiences from this experimental work.

## Data Availability

The raw data supporting the conclusions of this article will be made available by the authors, without undue reservation.

## Appendix

**Table A1:** Flanker test and reaction time.

	**Kichwa-Span-Bil**.		**Span.Monolinguals**	
	**Mean**	**St. dev**	**Mean**	**St. dev**
Flanker	7.13	0.815	8.09	0.77
Reaction time (ms)	5,288.3	1,182.5	3,931.2	687.75

**Table A2:** Flanker test for Quito and Guayaquil participants.

	**Kichwa-Span-Bil**.		**Span.Monolinguals**	
	**Mean**	**St. dev**	**Mean**	**St. dev**
Flanker	8.58	0.55	8.25	0.68

**Table A3:** Flanker test for Kichwa-Spanish bilinguals.

	**Mean**	**St. dev**
Flanker scores	7.708	0.68
